# Myelodysplastic Syndrome with t(1;7) Associated with Marked Dysmegakarypoiesis & Severe Thrombocytopenia: A Case Report and Review of the Literature

**DOI:** 10.1155/2012/167653

**Published:** 2012-04-12

**Authors:** Michael Gilbertson, Annabel Tuckfield, Surender Juneja

**Affiliations:** Department of Diagnostic Haematology, The Royal Melbourne Hospital, Parkville, VIC 3050, Australia

## Abstract

We present the case of a 70-year-old woman who had a bone marrow examination performed to investigate marked thrombocytopenia in the context of a recent history of metastatic glucagonoma. Surprisingly this identified marked dysmegakaryopoiesis and fulfilled diagnostic criteria for refractory cytopenia with multilineage dysplasia, with a relatively uncommon associated cytogenetic lesion t(1;7). We present the case and review the literature of this cytogenetic lesion.

## 1. Case Presentation

A 70-year-old woman was referred to our institution in June 2010 for further evaluation of moderate to severe pancytopenia. She was clinically relatively well with no recent infective symptoms or bleeding events. Her past history was noteworthy for metastatic glucagonoma diagnosed in June 2009, for which she had undergone surgical resection and radiolabelled octreotide therapy. She had an FDG-PET scan which confirmed metabolic remission in March 2010 and was scheduled to continue octreotide therapy at the time of referral however, the degree of thrombocytopenia limited the patient's tolerability of further octreotide. She also had insulin requiring type 2 diabetes as a result of the glucagonoma. 

**Figure 1:**
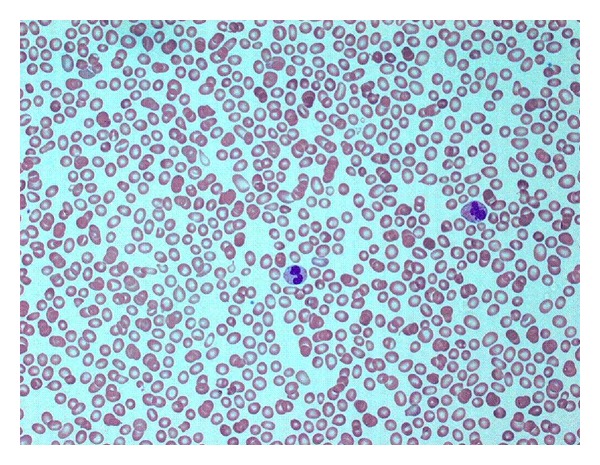
Mild red cell anisocytosis with macrocytosis and moderate numbers of tear drops. Mild granulocyte left shift and dysplastic changes (hyposegmented and hypersegmented neutrophils). Peripheral blood film. Wrights stain ×200.

Her initial full blood parameters (FBE) were Hb 107 g/L, MCV 105 fL, WBC 2.1 × 10^9^/L (Neutrophils 1.6 × 10^9^/L, Lymphocytes 0.3 × 10^9^/L), and Platelets 20 × 10^9^/L. The blood film showed mild red cell anisocytosis with macrocytosis and moderate numbers of tear-drop red cells (Figure 1). Granulocytes showed mild left shift and dysplastic changes (hyposegmented and hypersegmented neutrophils). Platelets were markedly reduced and showed marked anisocytosis with many large forms as well as abnormal granulation. Renal and liver functions were within normal limits. Previous FBEs performed at our institution in September 2008 revealed completely normal full blood parameters. Clinical examination was non-contributory.

**Figure 2:**
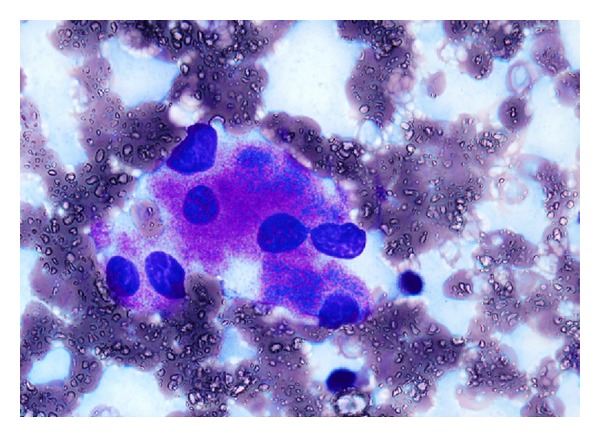
Marked megakaryocyte nuclear fragmentation, bone marrow aspirate, ICSH Romanovsky stain ×1,000.

**Figure 3:**
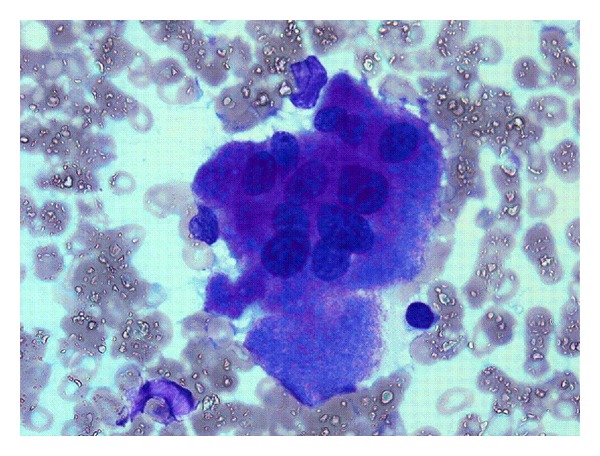
Marked megakaryocyte nuclear fragmentation, Bone marrow aspirate, ICSH Romanovsky stain ×1,000.

**Figure 4:**
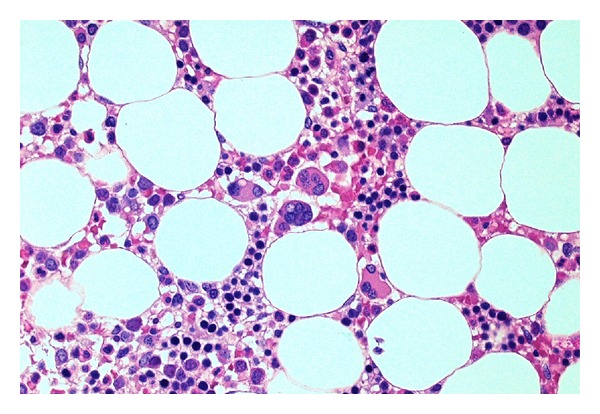
Marked megakaryocyte nuclear fragmentation in a normocellular marrow, bone marrow trephine H&E ×400.

**Figure 5:**
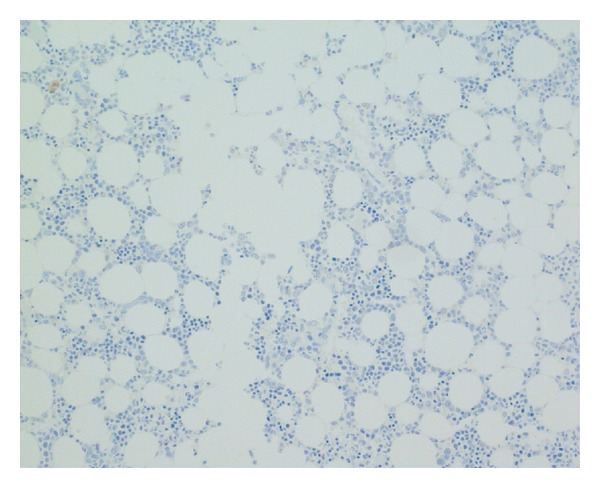
Negative cytokeratin immunohistochemistry, bone marrow trephine ×200.

**Figure 6:**
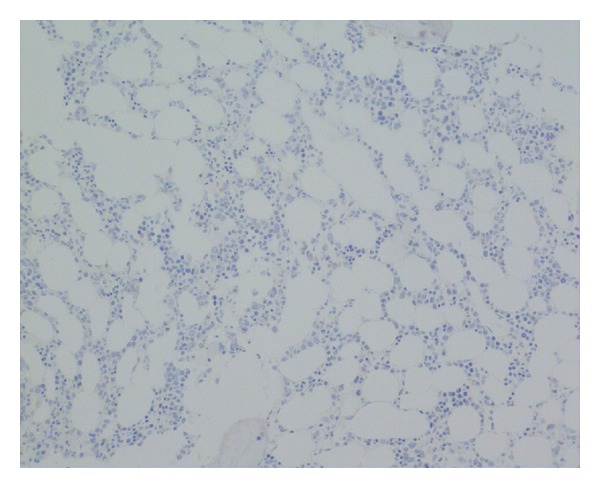
Negative synaptophysin immunohistochemistry, bone marrow trephine ×200.

**Figure 7:**
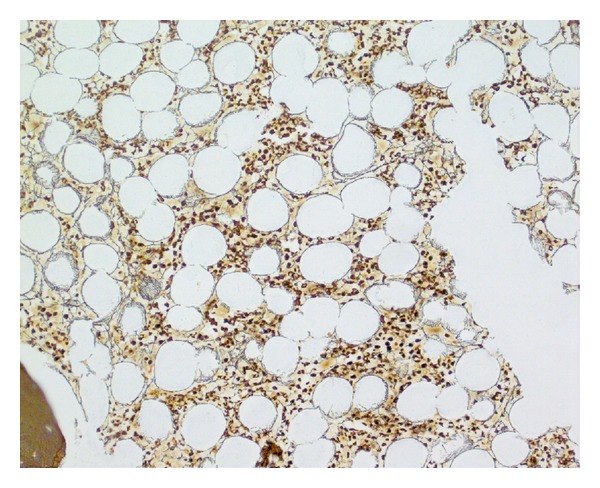
no increase in reticulin fibrosis, bone marrow trephine reticulin stain ×200.

**Figure 8:**
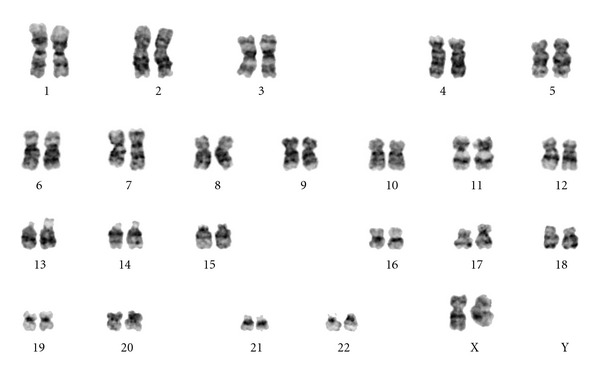
Representative conventional cytogenetics studies from the patient showing 46,XX,+1,der(1;7)(q10;p10). Image courtesy of A/Professor L Campbell, The Victorian Cancer Cytogenetics Service, St Vincents Hospital Melbourne.

Bone marrow examination was undertaken to exclude metastatic disease—of which there was none demonstrated on three heamatoxylin and eosin (H&E) levels and relevant neuroendocrine immunohistochemistry (cytokeratin and synaptophysin Figures 5 and 6, resp.). Surprisingly, the bone marrow showed mild increase in megakaryocyte numbers with marked megakaryocyte dysplasia; although they appeared normal in size, the nucleus of most megakaryocytes was markedly fragmented (Figures 2, 3 and 4) containing up to 20 separate nuclear fragments. There were also mild dysplastic features affecting the granulocyte and erythroid lineages and blasts comprised 3% of nucleated cells. There was no increase in reticulin fibrosis (Figure 7). Conventional cytogenetics studies revealed an unbalanced translocation 46,XX,+1,der(1;7)(q10;p10) in 12 out of 20 cells fully examined (Figure 8), which was further supportive evidence for a diagnosis of refractory cytopenia with multilineage dysplasia (RCMD) according to WHO 2008 diagnostic criteria [1]. FISH studies were not performed.

The patient received supportive care for her myelodysplastic syndrome with platelet transfusion for symptomatic bleeding events; however, no further hormonal therapy was able to be given to treat her glucagonoma. She remains alive with transfusion support at the time of this report, 4 months following the diagnosis of RCMD.

## 2. Discussion

The severity of the thrombocytopenia in this patient is unusual for MDS and probably reflects the degree of dysmegakaryopoiesis manifesting as nuclear fragmentation which was seen in all megakaryocytes. Megakaryocyte fragmentation may be seen in myelodysplastic syndromes (MDS) but in our experience is rarely as severe as in this patient.

Unbalanced translocations involving chromosome 1 and 7 are recognised cytogenetic abnormalities in MDS but are relatively uncommon, comprising 1–3% of de novo MDS cytogenetic abnormalities, as well as being present in 1-2% of acute myeloid leukaemias and 1% of myeloproliferative neoplasms [2]. Most patients with der(1;7) and MDS present with multilineage dysplasia and pancytopenia. As in our patient, a history of toxin exposure is identified in 40–50% of patients [3]. The prognosis is often poor with a high rate of progression to acute myeloid leukaemia and as such MDS with this cytogenetic subgroup constitutes a poor-risk on the International Prognostic Scoring System (IPSS), though this has recently been challenged, with some authors suggesting that it carries a more benign course than other abnormalities of chromosome 7 and should therefore be assigned an intermediate risk-karyotype [4].

Slovak et al. [5] retrospectively reviewed their single centre experience of 63 patients diagnosed with MDS and abnormalities of chromosome 7 on conventional cytogenetics between 1989 and 2008. Patients with complex cytogenetic abnormalities were excluded from analysis.

Specifically there were 51 cases with del (7q)/-7, and 12 had der(1;7). Patients with der(1;7) were more likely to be older and with more severe thrombocytopenia at diagnosis. Though there was a trend towards less inferior overall survival in patients with der(1;7), this did not reach statistical significance. There was no difference between patients with der(1;7) and risk of transformation to AML when compared with other isolated abnormalities of chromosome 7, and as such, the authors recommended that it carries a poor-risk karyotype in the IPSS; however, the small sample size makes definitive conclusions difficult.

Glucagonomas are rare neuroendocrine tumors of pancreatic Alpha-cell origin that secrete excessive amounts of glucagons, resulting in hyperglycaemia. Not surprisingly, as in the case of this patient, insulin requiring diabetes mellitus is a common presentation. Glucagonomas like other islet cell tumours may also secrete multiple other hormones including insulin, adrenocorticotropic hormone (ACTH), pancreatic polypeptide, parathyroid hormone (PTH) or substances with activity similar to PTH, such as gastrin, serotonin, vasoactive intestinal polypeptide (VIP), and melanocyte-stimulating hormone (MSH) [6]. Management for glucagonomas includes surgical resection for localised disease; however, for metastatic disease systemic therapy is required. Octreotide is a somatostatin analogue, which is useful to control symptoms. Recent clinical trials with radiolabelled somatostatin analogues ((90)Y-DOTATyr3-octreotide) have shown some promise with controlling metastatic disease [7, 8]. Although glucagonomas generally show slow-growth patterns, the prognosis for metastatic glucagonoma is poor, with a 5-year overall survival of 40%.

This case confirms the uncommon association between radiolabelled octreotide and development of a myelodysplastic syndrome [9], and is noteworthy for a rare cytogenetic abnormality, severe thrombocytopenia, and distinctive megakaryocyte morphology.
